# Isoliquiritigenin ameliorates caerulein‐induced chronic pancreatitis by inhibiting the activation of PSCs and pancreatic infiltration of macrophages

**DOI:** 10.1111/jcmm.15498

**Published:** 2020-07-17

**Authors:** Li‐Juan Wang, Lin He, Lu Hao, Hong‐Lei Guo, Xiang‐Peng Zeng, Ya‐Wei Bi, Guo‐Tao Lu, Zhao‐Shen Li, Liang‐Hao Hu

**Affiliations:** ^1^ Department of Gastroenterology Changhai Hospital The Second Military Medical University Shanghai China; ^2^ Shanghai Institute of Pancreatic Diseases Shanghai China; ^3^ Department of Gastroenterology & Endocrinology No. 969 Hospital of PLA Hohhot China; ^4^ Department of Gastroenterology First Affiliated Hospital Zhejiang University School of Medicine Hangzhou China; ^5^ Department of Digestive Diseases No. 900 Hospital of the Joint Logistics Team Fuzhou China; ^6^ Department of Gastroenterology Affiliated Hospital of Yangzhou University Yangzhou University Yangzhou China; ^7^ Jiangsu Co‐innovation Center for Prevention and Control of Important Animal Infectious Diseases and Zoonoses College of Veterinary Medicine Yangzhou China

**Keywords:** chronic pancreatitis, Isoliquiritigenin, macrophages, pancreatic fibrosis, pancreatic stellate cells

## Abstract

Chronic pancreatitis (CP) is characterized by persistent inflammation of the pancreas that results in progressive loss of the endocrine and exocrine compartment owing to atrophy and/or replacement with fibrotic tissue. Currently, the clinical therapeutic scheme of CP is mainly symptomatic treatment including pancreatic enzyme replacement, glycaemic control and nutritional support therapy, lacking of specific therapeutic drugs for prevention and suppression of inflammation and fibrosis aggravating in CP. Here, we investigated the effect of isoliquiritigenin (ILG), a chalcone‐type dietary compound derived from licorice, on pancreatic fibrosis and inflammation in a model of caerulein‐induced murine CP, and the results indicated that ILG notably alleviated pancreatic fibrosis and infiltration of macrophages. Further in vitro studies in human pancreatic stellate cells (hPSCs) showed that ILG exerted significant inhibition on the proliferation and activation of hPSCs, which may be due to negative regulation of the ERK1/2 and JNK1/2 activities. Moreover, ILG significantly restrained the M1 polarization of macrophages (RAW 264.7) via attenuation of the NF‐κB signalling pathway, whereas the M2 polarization was hardly affected. These findings indicated that ILG might be a potential anti‐inflammatory and anti‐fibrotic therapeutic agent for CP.

## 1 INTRODUCTION

Chronic pancreatitis (CP) is a pathological fibro‐inflammatory syndrome of the pancreas in individuals who develop persistent pathological responses to parenchymal injury or stress, with end result of endocrine and exocrine insufficiency and increased risk of pancreatic ductal adenocarcinoma.^1^ To date, treatment of patients with CP is mainly conservative or endoscopic, aiming at the removal of the underlying cause, the alleviation of pain, and the management of the exocrine and endocrine insufficiency, no specific therapies for prevention and suppression of the inflammatory damage or pancreatic fibrosis.^2^, ^3^ Therefore, identifying novel drugs for this disease would fill the unsatisfied demand to improve the quality of life and limit the medical costs associated with long‐term care of these patients.

Pancreatic stellate cells (PSCs) are the major effectors of pancreatic fibrosis. Following acute pancreas injury, PSCs differentiate into myofibroblast‐like cells with contractile, pro‐inflammatory and potent fibrogenic activities, as well as actively migrate and proliferate.^4^ Activated PSCs, expressing high level of α‐smooth muscle actin (α‐SMA), is capable of synthesizing and secreting amounts of ECM ingredients, such as collagens, fibronectin and connective tissue growth factor (CTGF) for tissue repair, and kinds of chemokines and cytokines that would facilitate the pancreatic infiltration of inflammatory cells to deal with necrotic tissue and apoptotic cells. However, if the cause of injury persists, ECM components and pro‐inflammatory factors accumulate to high levels, ultimately leading to advanced fibrosis and the onset of CP. Therefore, reversal or alleviation of pancreatic fibrosis by inhibiting PSCs activation might be a promising therapeutic approach for CP.

The mitogen‐activated protein kinases (MAPKs), including extracellular signal regulated kinase (ERK), c‐Jun N‐terminal kinase (JNK/SAPK) and p38 MAPK, function in protein kinase cascades that play an important role in the regulation of cell growth, differentiation, apoptosis and stress response.^5^ In pancreas, the MAPK signalling has been reported to be involved in pancreatic fibrosis by regulating PSCs functions, such as proliferation, migration and apoptosis of PSCs.^6^, ^7^, ^8^ R. Jaster and colleagues reported that PSC proliferation in response to PDGF was mediated by the ERK1/2 pathway.^9^ Other researches indicated that inhibition of either JNK1/2 or p38 MAPK phosphorylation could block the activation of PSCs.^10^, ^11^

Besides PSCs, recent studies also highlighted the function of macrophages as master regulators of pancreatic inflammation, fibrosis and tumorigenesis.^12^, ^13^ It has been reported that the infiltration of macrophages increased threefold to fourfold in CP pancreas compared to normal ones.^14^ Two known macrophage subpopulations, classically activated macrophages (M1, upon exposure to interferon‐γ (IFN‐γ) and/or lipopolysaccharide (LPS), play an important role in host defence and anti‐tumour immunity) and alternatively activated macrophages (M2, induced by IL‐4/IL‐13, play a critical role in fibrosis, promote wound healing, dampen inflammation and tumorigenesis), can be differentiated by specific surface markers and protein expression profiles.^15^, ^16^ Distinct macrophage populations contribute critical activities towards the initiation, maintenance and resolution phase of fibrosis,^17^ though much remains unclear.

Isoliquiritigenin (ILG), a simple chalcone‐type flavonoid isolated from licorice roots, has been reported to exhibit anti‐oxidative, anti‐inflammatory, oestrogenic and hepatoprotective properties.^18^, ^19^, ^20^ It is reported that ILG could suppress adipose tissue inflammation and attenuate high fat diet–induced adipose tissue fibrosis by targeting innate immune sensors.^21^ S. Wu *et al* reported that ILG inhibited IFNγ‐inducible genes expression in hepatocytes through down‐regulating the activation of ERK, JNK and PI3K/Akt signalling pathways, but not P38 MAPK pathway.^22^ Particularly, recent study by X. Liu and colleagues has showed that ILG is effective in alleviation the severity of acute pancreatitis (AP) via inhibition of oxidative stress and modulation of the Nrf2/HO‐1 pathway,^23^ which prompted us to investigate whether ILG is functional in protecting against CP and the underlying mechanism.

In this study, we analysed the influence of ILG on the progression of caerulein‐induced murine CP. Results showed that ILG significantly alleviated pancreatic fibrosis and inflammation by inhibiting both PSCs activation and macrophages pancreatic infiltration. Further in vitro RNA‐seq analysis and validation experiments in hPSCs confirmed that ILG suppressed the activation of PSCs via negative regulating the ERK1/2 and JNK1/2 activities and relevant signalling pathways. In addition, ILG also notably restrained the M1 polarization of RAW 264.7 macrophages through NF‐κB pathway. These findings suggested that ILG may be a potential therapeutic agent for mitigation of both pancreatic fibrosis and inflammation in CP.

## 2 MATERIALS AND METHODS

### 2.1 Reagents

ILG (Isoliquiritigenin, CAS: 961‐29‐5) was purchased from Aladdin (Aladdin Bio‐Chem Technology Company, Shanghai, China). Caerulein (Cae) and dimethyl sulfoxide (DMSO) was obtained from Sigma‐Aldrich. Recombinant human TGF‐β1 and recombinant mouse IL‐4 were obtained from R&D Systems (Minneapolis, MN, USA). Lipopolysaccharide (LPS) was purchased from InvivoGen (InvivoGen, France). Sodium carboxymethyl cellulose (Na‐CMC) was obtained from Sangon Biotechnology (Shanghai, China). Early Apoptosis Detection Kit (#6592) was obtained from Cell Signaling Technology (Danvers, Massachusetts, USA).

### 2.2 Mice

**FIGURE 1:**
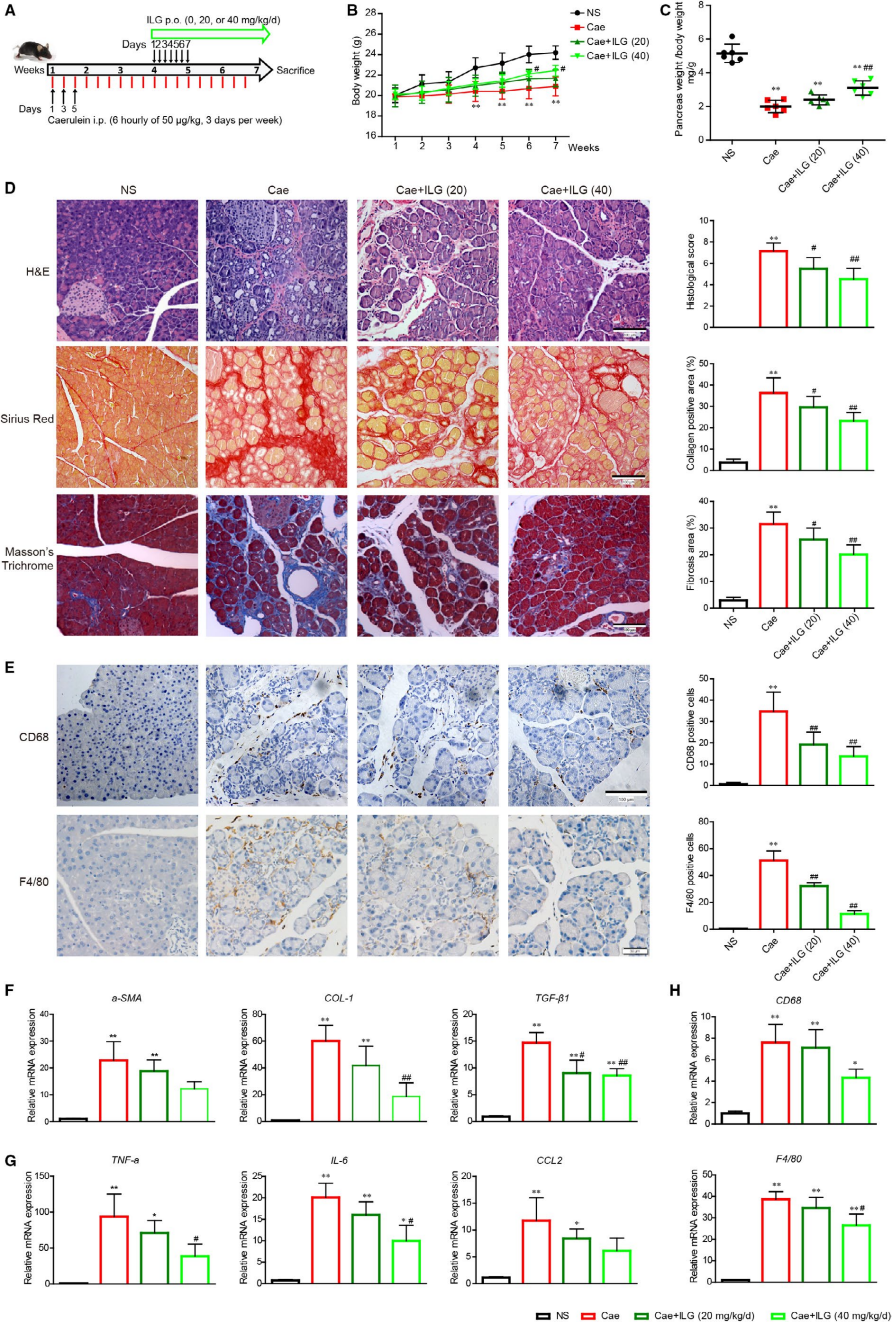
Inhibitory actions of ILG against pancreatic fibrosis and inflammation. The flow diagram of caerulein‐induced experimental chronic pancreatitis (CP) and the intervention with ILG (A). The bodyweights of each group were monitored weekly over the experimental procedure (B). The relative pancreas weights were recorded at the end of the experiment (C). The histologic features of pancreases were exhibited by H&E, Sirius red and Masson's trichrome staining (D). Immunohistochemistry (IHC) staining for CD68 and F4/80 (brown, surface markers of macrophages) was performed on pancreatic paraffin sections (E). The histologic scores and quantitative analysis are presented, respectively, on the right side. Representative sections of pancreases were from at least five mice per group. qRT‐PCR analysis of the mRNA transcription of fibrogenic (F), pro‐inflammatory genes (G) and surface marker genes of macrophages (H). The data were expressed as relative fold changes over the values of the NS group and were all presented as mean ± SD of at least three independent experiments. *P < .05, **P < .01 compared with the NS group; #*P* < .05, ##*P* < .01 compared with the Cae group

C57BL/6 male mice (19‐21 g, 6‐7 weeks old) were purchased from Lingchang Biotechnology Co., Ltd (Shanghai, China). All mice used throughout the study were housed in humidity‐ and temperature‐controlled room with 12‐hours light/dark cycle. Murine experiments were taken place in the specific pathogen‐free (SPF) animal house of Changhai Hospital (Naval Medical University, Shanghai, China). Mice were randomly assigned into 4 groups (6 mice for each group): NS, Cae, Cae + ILG (20 mg/kg) and Cae + ILG (40 mg/kg). To generate experimental CP, 6 hourly intraperitoneal (i.p.) injections of caerulein (Cae, 50 μg/kg) were performed in C57BL/6 male mice, 3 days per week, for a total of 6 weeks. Mice were killed 5 days after the last caerulein injection.^24^ Upon Cae injection, mice in Cae group and Cae + ILG group were orally administered with either vehicle solution (0.5% Na‐CMC) or ILG (20, 40 mg/kg/day) solution, respectively, from the first day of the 4th week till the day before killing (Figure 1A). In control group, mice received 0.5% Na‐CMC vehicle solution and injections of normal saline (NS) in lieu of caerulein. Ethical approval for all experimental procedures had been obtained from the Committee of Changhai Hospital, Naval Medical University, Shanghai, China.

### 2.3 Cell lines and cell culture

Human pancreatic stellate cells (hPSCs) were obtained as a gift from Prof. Logsdon CD (Department of Cancer Biology, University of Texas MD Anderson Cancer Centre, Houston, Texas, USA) and cultured in Dulbecco's modified eagle medium (DMEM, Hyclone Laboratories), supplemented with 15% foetal bovine serum (FBS, Gibco, USA) and 1% penicillin/streptomycin (Gibco, USA), and the medium was replaced every other day.^24^ RAW 264.7 cells, a mouse peritoneal macrophage cell line, were obtained from American Type Culture Collection (ATCC, VA, USA) and were cultured in DMEM (supplemented with 10% FBS, and 1% penicillin/streptomycin). All cells were maintained at 37°C in a humidified atmosphere of 5% CO_2_.

For pharmacologic efficacy analysis and mechanistic studies, hPSCs, after reaching about 70% confluence, were treated with different dose of ILG (0, 5, 10 or 20 μM, dissolved in DMSO) with or without TGF‐β1 (2 ng/mL) pre‐induction for 1 hours. Cells were harvested at various time points and then subjected to quantitative real‐time PCR (qRT‐PCR), Western blot (WB) or immunofluorescent (IF) staining as described below. RAW 264.7 cells were pre‐treated with either LPS (500 ng/mL) for 1 hours or recombinant mouse IL‐4 (20 ng/mL) for 24 hours, and followed by co‐administration with ILG (0, 5, 10 or 20 μM) for another 24 hours. Cells were then subjected to qRT‐PCR or WB as described below.

### 2.4 Quantitative real‐time PCR (qRT‐PCR)

Total RNA was extracted from cells or mice pancreas using TRIzol reagent (Invitrogen, USA), and then 1 μg of total RNA was reverse‐transcribed into cDNA utilizing the RevertAid First Strand cDNA Synthesis Kit (Thermo Scientific, USA). qRT‐PCR was performed on a Light Cycler^®^ 480 II System (Roche, Sandhofer, Germany) using Hieff^TM^ qPCR SYBR^®^ Green Master Mix (YEASEN Biotechnology, Shanghai, China) as per the manufacturer's instruction. Relative mRNA expression levels were calculated using the 2^‐△△CT^ method and each experiment was repeated at least three times. Human *GAPDH* or mouse *Gapdh* was used as internal control gene. The primer sequences are listed in Table S1.

### 2.5 Western blotting (WB)

Cell lysates were prepared on ice using RIPA lysis buffer (Beyotime Biotechnology, China). Total protein was purified by centrifugalizing and the concentration was determined by the BCA kit (Thermo Fisher Scientific, Illinois, USA). Equal amounts of lysates containing 20‐50 µg total protein were subjected to standard WB procedure.^24^ Primary antibodies used in this study are described in Table S2. Horseradish peroxidase‐conjugated secondary antibodies (Jackson ImmunoResearch, West Grove, PA) were used at 1:5000‐1:10 000. β‐actin was used as internal control. Densitometry analysis for each target protein was performed using ImageJ software.

### 2.6 Histology and immunohistochemistry (IHC)

Pancreas, lung, liver, kidney and heart from killed mice were immediately fixed in 4% paraformaldehyde (PFA) and fixed for at least 48 hours at 4°C. Then, tissues were embedded in paraffin, and sections were taken by 5‐μm and mounted on slides for staining. Haematoxylin and eosin staining (H&E) was conducted for histopathological evaluation. Sirius Red staining (ab150681, Abcam) and Masson's Trichrome staining (Connective Tissue Stain) (ab150686, Abcam) were performed according to the instructions, respectively, for fibrotic evaluation. IHC staining was performed for infiltrating macrophages by CD68 and F4/80 antibodies, respectively. Semiquantitative analysis of the staining was performed using ImageJ software.

For CP injury, the severity was double‐blindly graded in scores by a pathologist unaware of the origin of the specimens. Briefly, the scores were graded as described before ^25^: inflammatory cell infiltration (0, absent; 1,<5%; 2, <10%; 3, <50%; 4, ≥50%), acinar atrophy (0, absent; 1, minimal; 2, moderate; 3, major or severe), and fibrosis (0, absent; 2, only within areas; 4, diffuse). For all the scores, 5 pancreas sections were randomly selected from each mouse, and 6 randomly chosen microscopic fields were examined for each tissue section.

### 2.7 Cell viability assay

Cells viability was determined by the Cell Counting Kit‐8 (KeyGEN, Shanghai, China). The hPSCs and Raw 264.7 cells were seeded on a 96‐well plate at a density of 1 × 10^4^ cells per well with various concentrations of ILG for 24 h. Then, the test solution (10 μL) was added to the medium and incubated for another 3 hours at 37°C. The optical density values of each well were measured at 450 nm and the results from different groups were compared.

### 2.8 Immunofluorescence (IF)

For immunofluorescent staining, hPSCs were cultured on chamber slides and treated with ILG (20 μM) or TGF‐β1 (2 ng/mL) or both for a total of 24 hours. Then, cells were fixed with 4% PFA and permeabilized with 0.1% Triton X‐100 and then blocked with 5% BSA and incubated with primary antibodies overnight at 4°C in a dark humidity chamber. After incubated with the secondary antibody of either Alexa Fluor 594 AffiniPure goat anti‐rabbit or goat anti‐mouse IgG (H + L) (1:500; YEASEN, China), cells were counterstained with 4, 6‐diamidino‐2‐phenylindole (DAPI, Invitrogen, USA) for nuclear visualization. Cells were observed using the fluorescence microscope, and photomicrograph images were captured using the same exposure time.

### 2.9 Wound‐healing assay

The well‐growing hPSCs were collected and inoculated into a 6‐well plate at a density of 5 × 10^5^ cells/well. After cells adhered to the plate, original culture medium was replaced by serum‐free culture medium for 24‐hours incubation. The ‘scratch’ wound was created using a 100‐µL sterile pipette tip. Then, fresh serum‐free culture medium supplemented with control DMSO, ILG (5, 10, 20 μM) or TGF‐β1 (2 ng/mL) were added for further 24 hours. Cell migration was evaluated at 0, 12 and 24 hours and images were taken by an inverted microscope and analysed using ImageJ software.

### 2.10 Transwell assay

HPSCs (2 × 10^4^) were seeded into a Falcon^®^ Cell Culture Inserts (Corning, NY, USA) with a pore size of 8.0 μm in a 12‐well plate. The upper chamber contained 100 μl serum‐free culture medium plus DMSO, ILG (5, 10, 20 μM) or TGF‐β1 (2 ng/ml), and 500 μL culture medium (contained 10% FBS) were added into the lower 12‐well plates. After 24 hours of incubation, cells in the upper chamber were wiped, and cells at the bottom of the membrane were stained with crystal violet and then imaged and counted in 5 randomly chosen fields.

### 2.11 RNA‐seq analysis

Total RNA was isolated from hPSCs treated with DMSO or ILG (20 μM) for 4 hours using the mirVana miRNA Isolation Kit (Ambion) following the manufacturer's protocol. The RNA library was sent for sequencing on Illumina sequencing platform (Illumina HiSeq X Ten) using next‐generation sequencing analysis. The sequencing data were analysed to screen for differentially expressed genes (DEGs).^24^ Based on DEGs, deep‐sequencing analysis was performed including Gene Ontology (GO) enrichment and KEGG pathway enrichment analyses (OE Biotech., Shanghai, China).

### 2.12 Serum biochemical indices

The serum alanine aminotransferase (ALT), total cholesterol (T‐CHO), triglyceride (TG) and glucose (Glu) levels were measured by corresponding colorimetric assay kit (Elabscience, Wuhan, China) according to their instructions.

### 2.13 Statistical analysis

Data were analysed by unpaired Student's t test for comparison between two experimental groups, one‐way ANOVA followed by Tukey's multiple comparison tests or two‐way ANOVA for inter‐group differences among 3 groups or more using the GraphPad Prism software (version 5.01). The normality was checked using the Kolmogorov‐Smirnov test. The results were presented as mean ± standard deviation (SD) of at least three independent experiments. *P* value of < .05 is accepted as statistically significant.

## 3 RESULTS

### 3.1 ILG attenuates caerulein‐induced pancreatic fibrosis and inflammation in vivo

In previous study, ILG was reported to show protective effects on Cae‐induced AP in mice,^23^ which prompted us to investigate whether ILG is effective in protecting against CP. As shown in Figure 1A, in addition to Cae injection, mice were orally administered with ILG (0, 20 and 40 mg/kg/day) from the 4th week till the day before killing. The bodyweights of mice in each group were measured every week. A significant lower in bodyweight was observed in Cae‐induced CP mice compared to normal saline (NS)‐treated group from the 4th week and developed along with the experiment progress, which was notably alleviated by additional ILG (40 mg/kg/day) administration (Figure 1B). Mice undergoing repetitive treatment with Cae also displayed smaller size of the pancreas relative to bodyweight due to acinar cell loss, which was partially improved by ILG dose‐dependently (Figure 1C), indicating that ILG is beneficial for preventing the loss of bodyweight and pancreatic parenchyma induced by repetitive injections of Cae.

To further validate the effect of ILG on the progression of CP, we checked the histopathological alterations of pancreatic tissue in mice of each group and performed H&E staining, Sirius red and Masson's trichrome staining on pancreatic tissue sections (Figure 1D). Pancreas from Cae group revealed severe morphologic signs of CP with acinar cell atrophy, ducts dilatation, extensive ECM collagen accumulation and immune cells infiltration, all of that were markedly relieved with ILG administration. Staining for collagens and ECM ingredient (Figure 1D) and IHC staining for macrophages by CD68 and F4/80 antibodies (Figure 1E) in pancreatic tissues suggested that both the activation of PSCs and pancreatic infiltration of macrophages were significantly increased in CP mice and were notably reduced by additional ILG administration.

Next, qRT‐PCR assays were done using pancreatic tissues to confirm the influence of ILG on the expression of fibrogenic and pro‐inflammatory genes. The mRNA levels of major fibrogenic genes (*α‐SMA*, *Col1α1 (COL‐1)* and *TGF‐β1*), pro‐inflammatory genes (*TNF‐α*, *IL‐6* and *CCL2*) and the macrophage cell surface marker genes (*CD68, F4/80*) were all significantly increased in Cae group and were partially alleviated in ILG treatment groups (Figure 1F‐H). These results together indicated that ILG has a potential role in meliorating the pancreatic fibrosis and inflammation in Cae‐induced CP.

Besides, ILG treatment hardly affects the histologic structure of organs such as liver, lung, spleen and kidney (Figure S1A), and the serum biochemical indices, like serum ALT, TG, T‐CHO and Glu levels were all not obviously changed (Figure S1B), indicating that ILG had no obvious systemic toxicity.

### 3.2 ILG inhibits the activation of PSCs in vitro

**FIGURE 2:**
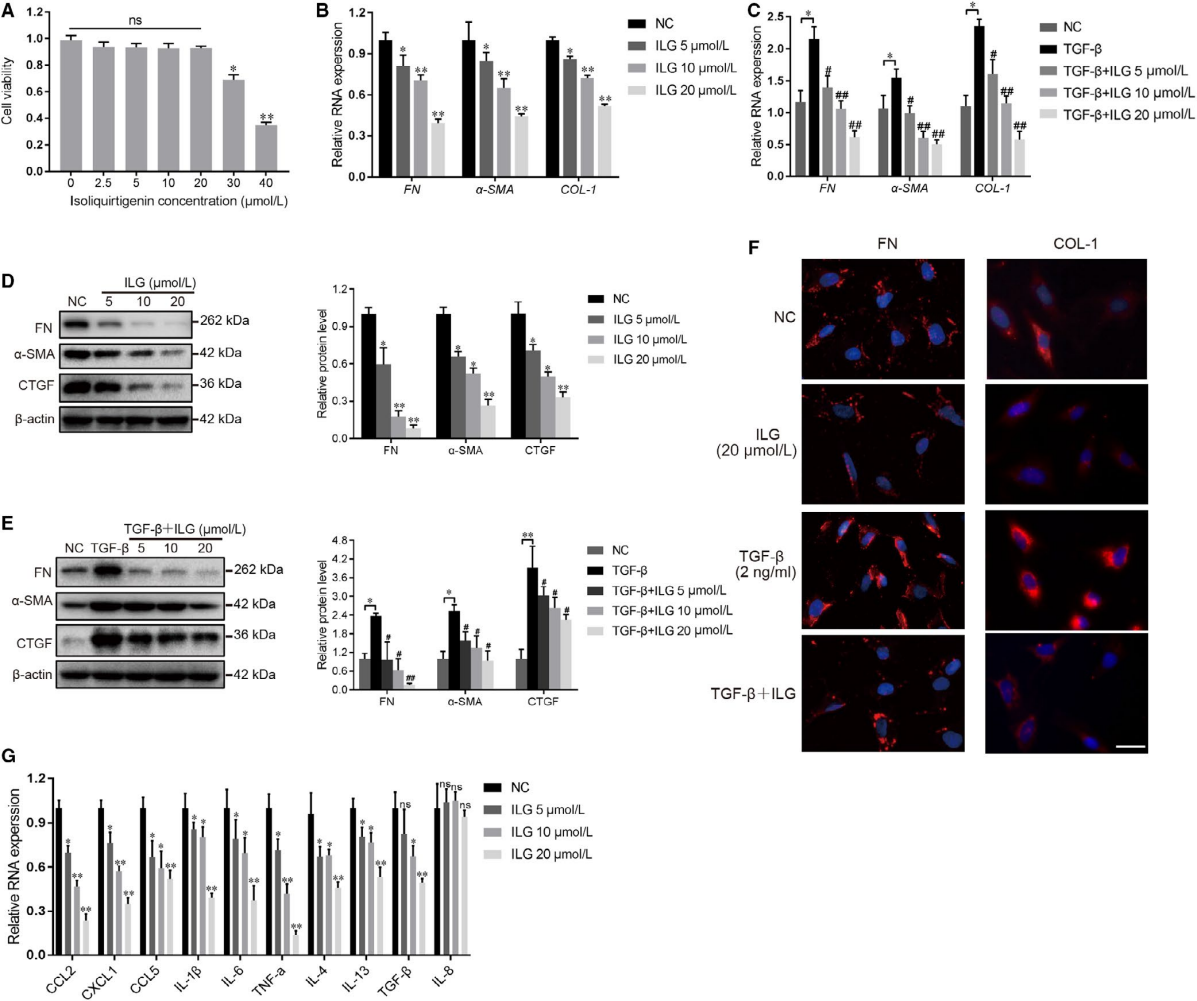
ILG inhibits the activation of hPSCs. (A) The cell viability of hPSCs was measured by cell counting kit‐8 (CCK‐8) assay after treatment with different doses of ILG for 48 h. qRT‐PCR (B and C) and WB (D and E) were used to analyse the effect of ILG on the activation of hPSCs in the presence or absence of TGF‐β1 pre‐induction. The band signals were assessed using GeneTools software and statistically presented in the histogram on the right. β‐actin was used to normalize the intensity of band signals. (F) Immunofluorescence (IF) staining (red) for fibronectin (FN) and collagen 1 (COL‐1) were performed on hPSCs with indicated ILG and/or TGF‐β1 treatment, and the nuclei were stained with DAPI (blue). Scale bar = 100 μm. (G) The expression of inflammatory chemokine and cytokine genes (*CCL2、CXCL1、CCL5、IL‐1β、IL‐6、TNF‐α、IL‐4、IL‐13、TGF‐β1* and *IL‐8*) of hPSCs with different dose of ILG treatment were quantified by qRT‐PCR. All data were presented as mean ± SD of at least three independent experiments. *P < .05, **P < .01 compared with the NC group; #*P* < .05, ##*P* < .01 compared with the TGF‐β1 group; ns: *P* ＞ .05

Previous studies demonstrated that activated PSCs which contribute to the accumulation of ECM proteins lead to the onset and development of fibrosis in CP. Thus, we next evaluated the effect of ILG on the activation of hPSCs. First, cell viability of hPSCs treated with different doses of ILG (0‐40 μM) was measured by CCK‐8 assay. As shown in Figure 2A, ILG at the dose of 30 and 40 μM significantly decreased the cell viability of hPSCs. Thus, the dose of 20 μM was chosen as the maximum dose in following studies.

The activation of PSCs is characterized by the expression of many fibrogenic genes including *α‐SMA, COL‐1, FN* and *CTGF*, all of which would be dramatically up‐regulated by exogenous TGF‐β1 induction. To evaluate the influence of ILG on PSCs activation, hPSCs were treated with ILG and in either presence or absence of TGF‐β1 (2 ng/ml). Subsequent qRT‐PCR and WB results indicated that ILG significantly reduce the mRNA transcription (Figure 2B and C) and protein level (Figure 2D‐E) of these fibrogenic genes in both conditions, and in a dose‐dependent manner. Meanwhile, IF staining assays further confirmed that the expression of fibronectin (FN) and collagen 1 (COL‐1) were both notably decreased by ILG in either presence or absence of TGF‐β1 induction (Figure 2F).

In addition, activated PSCs can produce kinds of chemokines (CCL2, CCL5 and CXCL1) and cytokines (IL‐1β, IL‐6, TNF‐α, IL‐4, IL‐13 and TGF‐β1) to facilitate the tissue infiltration of immune cells, which were also suppressed by ILG dose‐dependently (Figure 2G), indicating an inhibitory role of ILG on the inflammatory response in CP, whereas IL‐8, also known as CXCL8 (a potent neutrophil chemotactic factor), was hardly affected by ILG treatment, which may be due to its low expression in hPSCs.

### 3.3 ILG inhibits the proliferation of hPSCs while inducing its apoptosis

**FIGURE 3:**
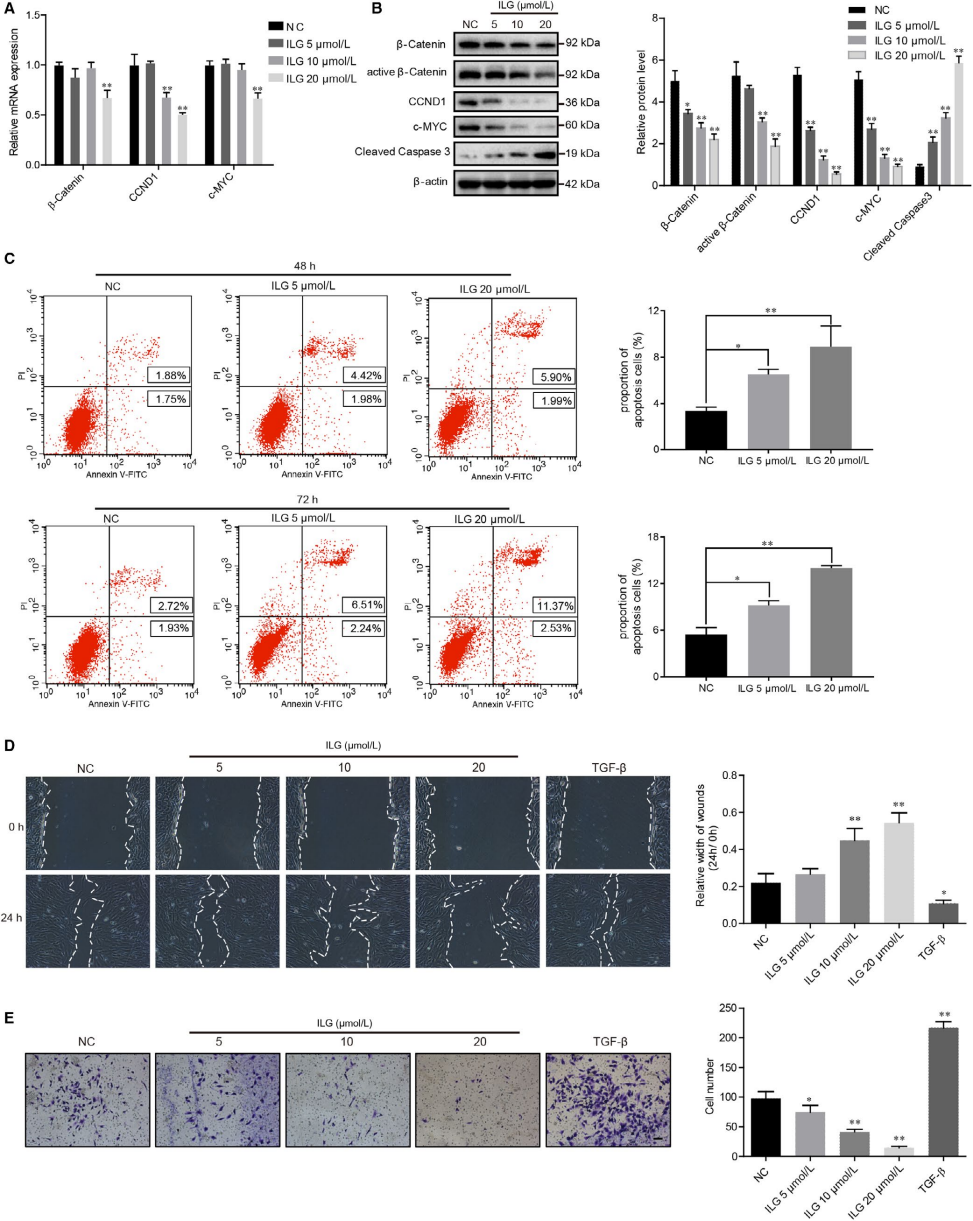
ILG inhibits the proliferation and migration of hPSCs, and meanwhile induces its apoptosis. The mRNA and protein level of proliferative genes like β‐Catenin, CCND1 and c‐MYC were detected by qRT‐PCR (A) and WB (B) assays in hPSCs treatment with indicated doses of ILG for 24 h. (C) Annexin‐V/PI bivariate flow cytometric analysis were used to analyse apoptosis of hPSCs (late apoptosis + early apoptosis) after ILG treatment (0, 5, 20 μM) for 48 or 72 h. (UL: necrosis; UR: late apoptosis; LL: live; LR: early apoptosis) The cell migratory capacity was measured by wound‐healing assay (D) and Transwell cell migration assay (E) in hPSCs. Images were captured after treatment with ILG (0, 5, 10, 20 μM) or TGF‐β1 (2 ng/mL) for 24 h. The width of wounds was measured using ImageJ and statistically presented in the histogram. The numbers of cells that migrating through the transparent polyethylene terephthalate membrane were counted and calculated (at least 6 random areas for each group). All data are expressed as mean ± SD of three independent repeated experiments. Scale bar = 100 μm. *P < .05, **P < .01 compared with the NC group

The Wnt/β‐Catenin pathway plays an important role in cellular proliferation and differentiation, and abnormal activation of Wnt/β‐Catenin signal was observed in CP.^26^ Thus, we checked the influence of ILG on Wnt/β‐Catenin pathway in hPSCs and found that ILG inhibited the protein expression and activity of β‐Catenin. The expression of downstream proliferative genes such as *CCND1 (cyclin D1)* and *c‐MYC* were also suppressed by ILG dose‐dependently at both mRNA and protein level (Figure 3A and B). Meanwhile, the cleaved Caspase 3, a key apoptotic marker, was significantly increased by ILG, suggesting a promotive effect of ILG on the apoptosis of hPSCs. We next analysed the effect of ILG on hPSCs by Annexin‐V/PI bivariate flow cytometry, which further confirm that ILG significantly promoted the apoptosis of hPSCs (Figure 3C).

### 3.4 ILG inhibits the migration of hPSCs

The capacity of cell migration is another biological aspect of PSCs that related to its activation status, and the augment of PSCs during CP may be due to increased local proliferation and/or migration of cells from adjacent areas.^27^ Hence, we performed wound‐healing assays (Figure 3D) and transwell assays (Figure 3E) to evaluate the effects of ILG on the migratory capacity of hPSCs. In the wound‐healing assays, compared to control group, TGF‐β1 treatment notably decreased the average wound width at 24 hours, whereas ILG treatment led to a significant increase in the wound width dose‐dependently (Figure 3D). While in the transwell assays, as showed in Figure 3E, compared to control group, TGF‐β1 stimulation significantly increased the number of hPSCs passing through the membrane, and ILG treatment significantly decreased the number in a dose‐dependent manner.

### 3.5 RNA‐seq analysis suggests a regulative role of ILG on the MAPK activity

**FIGURE 4:**
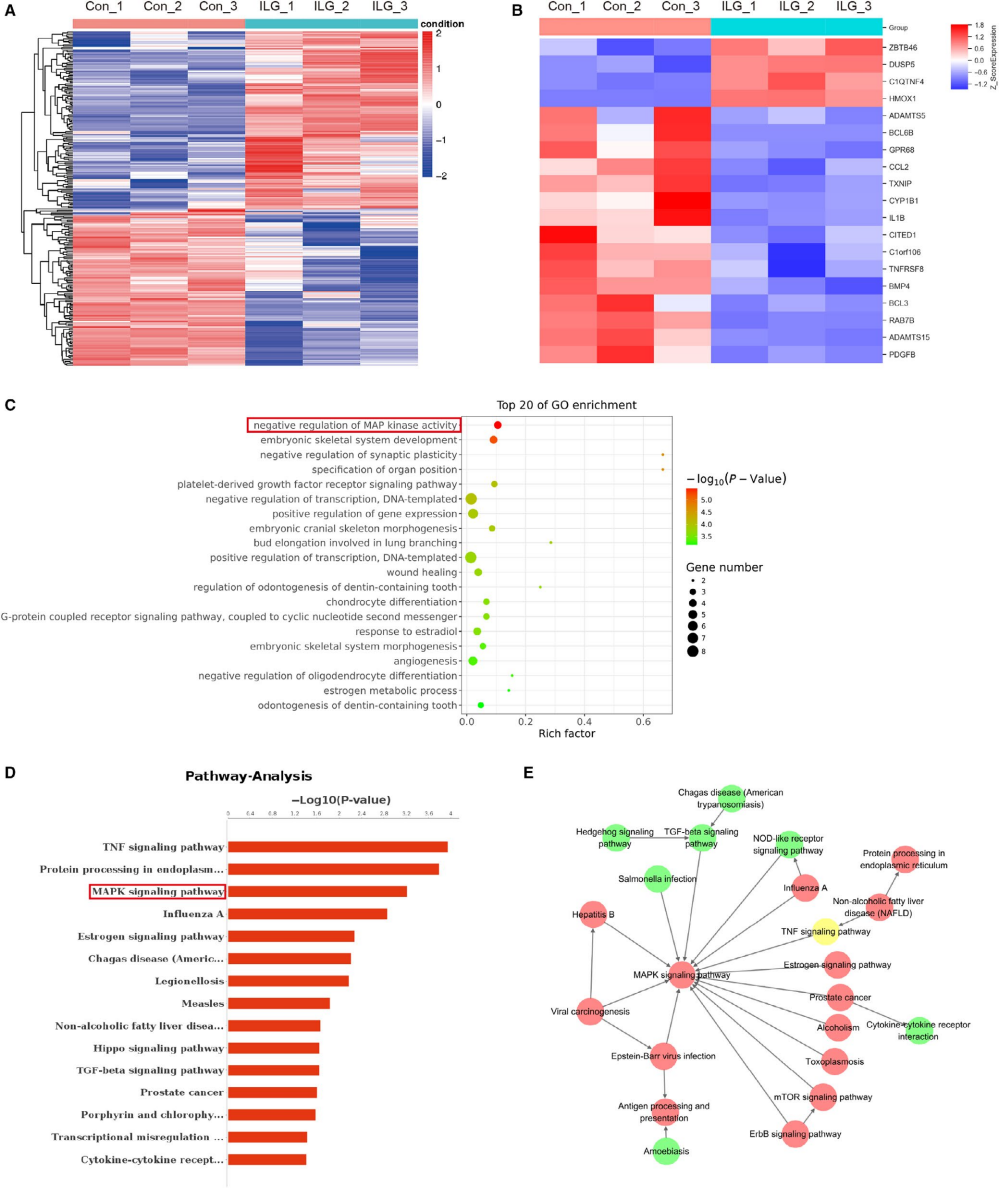
RNA‐seq analysis in hPSCs with or without ILG treatment. Hierarchical cluster analysis of DEGs was performed to explore transcripts expression pattern. According to the screening criteria (*P* value < .05 and fold Change > 2 or fold Change < 0.5), a total of 238 differentially expressed genes (DEGs) were identified between ILG treatment group and control group. The hierarchical clustering heat map of all DEGs (A) and part of the pancreatic fibrogenic and pro‐inflammatory–related DEGs (B) were depicted. The top 20 GO term (C) and pathway analysis (D) analysis of DEGs was performed using R based on the hypergeometric distribution. (E) The pathway interaction net of the DEGs was analysed and depicted, in which the up‐ (red circle) and down‐regulated (green circle) pathways were indicated

To further analysing the molecular mechanism of ILG on PSCs, transcriptomic analysis (RNA‐seq) was performed on hPSCs treated with ILG (20 μM, 4 hours). A total of 238 differentially expressed genes (DEGs) were identified between ILG group and control group, according to the screening criteria (*P* value < .05 and fold Change > 2 or fold Change < .5), consisting of 110 up‐regulated genes and 128 down‐regulated genes. Figure 4A and B showed the hierarchical clustering heat map of all DEGs and a part of fibrogenic and pro‐inflammatory DEGs, respectively. Pancreatic fibrogenic genes, ECM constituents and inflammation‐related genes such as IL‐1β, ADAMTS5, ADAMTS15, BMP4, Clorf106, CCL2 and PDGFB were all down‐regulated in ILG group, whereas the HMOX1 (haeme oxygenase 1, HO‐1), key protein of the Nrf2/HO‐1 anti‐oxidant pathway, was significantly up‐regulated by ILG, which was consistent with previous studies.^23^, ^28^ The top 20 GO enrichment analysis (Figure 4C) and pathway analysis (Figure 4D) showed that the MAPK signalling pathway might be the key pathway response to ILG treatment. The pathway interaction net also indicated a central role of the MAPK signalling pathway in regulating the proliferation and activation of PSCs by ILG (Figure 4E).

It is worth noticing that DUSP5, a dual specificity phosphatase, was notably up‐regulated by ILG (Figure 4B). The DUSPs are a family of protein phosphatases that are able to recognize both phospho‐Ser/Thr and phospho‐Tyr residues in substrates.^29^ A subgroup (DUSP‐MKPs, 10 members) of this family which contain an N‐terminal MAPK‐binding domain are function as MAPK phosphatases.^30^ Each of them has their own substrate specificity and physiological functions in negative regulation of MAPKs activities. For example, DUSP5 specifically binds and dephosphorylates ERK1/2, but not JNK1/2 and p38 MAPK; while DUSP10/MKP5 binds and inactivates JNK1/2 (and p38 MAPK in vitro.), but not ERK1/2.^31^ Thus, it is suggesting that ILG might inhibit the activation of PSCs through regulating the MAPKs’ activities.

### 3.6 ILG suppresses the activity of MAPKs through inhibiting the PDGFR pathway and promoting the expression of DUSP5 and DUSP10

**FIGURE 5:**
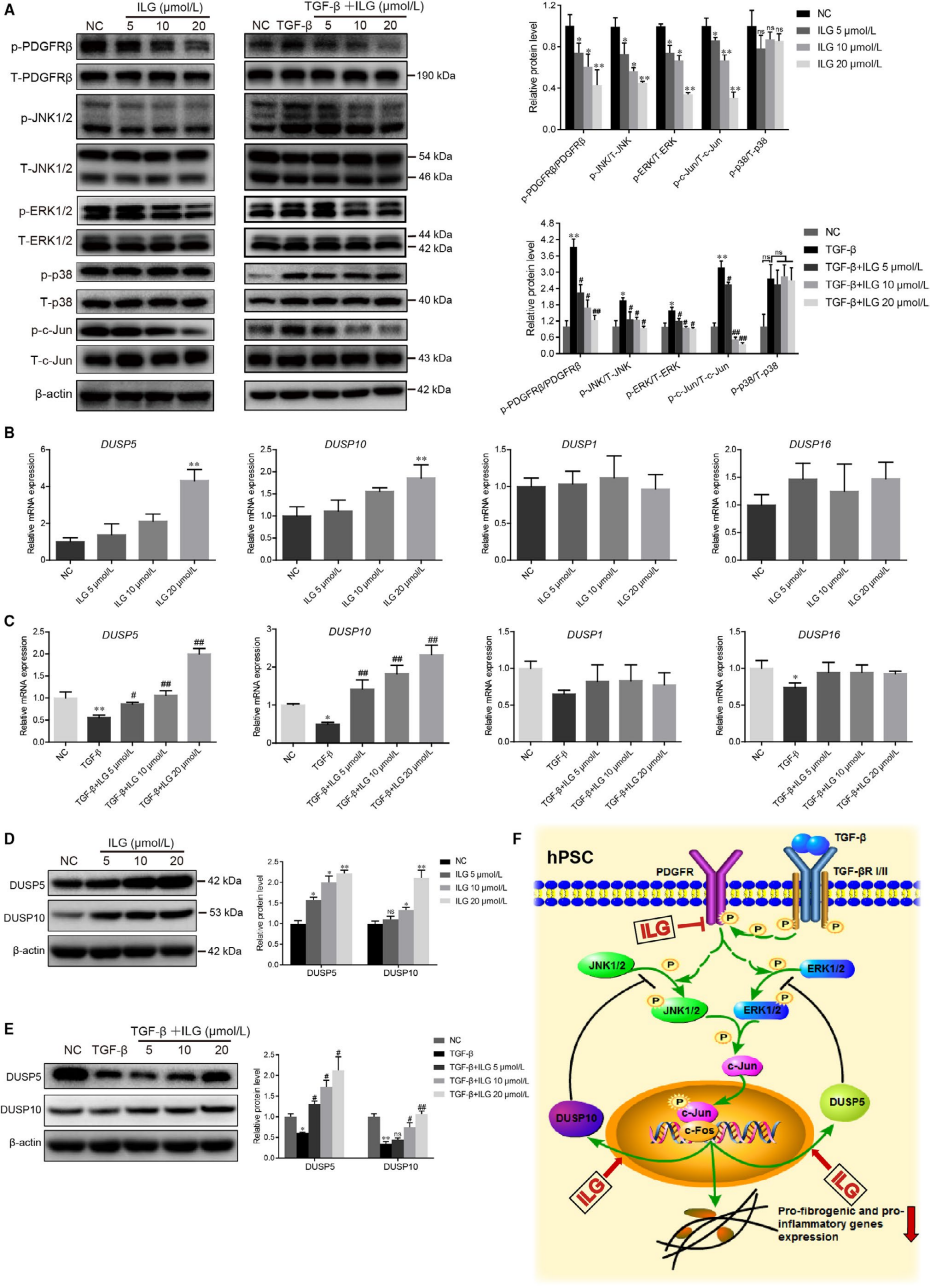
ILG inhibits PSCs activation by regulating the MAPK signalling pathway. (A) WB analysis of the protein expression and activity levels of key factors from the MAPK signalling pathway (PDFGRβ, JNK1/2, ERK1/2, p38 and c‐Jun) in hPSCs with indicated ILG (left panel) or TGF‐β1 plus ILG (right panel) treatment for 4 h. The band signals were assessed using GeneTools software and statistically presented in the histogram on the right. (B and C) The mRNA expression level of *DUSP5*, *DUSP10*, *DUSP1* and *DUSP16* were analysed by qRT‐PCR in hPSCs treated with ILG alone (B) or TGF‐β1 plus ILG (C). The protein levels of DUSP5 and DUSP10 in hPSCs treated with ILG alone (D) or TGF‐β1 plus ILG (E) were analysed by WB and statistically presented on the right. All data were presented as mean ± SD of at least three independent experiments. *P < .05, **P < .01 compared with the control group; #*P* < .05, ##*P* < .01 compared with the TGF‐β1 group; ns: *P* ＞ .05. (F) A schematic diagram illustrating the underlying mechanism by which ILG inhibits the expression of fibrogenic and pro‐inflammatory genes in hPSCs

To further validate the regulation of ILG on MAPKs activities, WB assays using antibodies against either phosphorylated or total three MAPKs (including ERK1/2, JNK1/2 and p38 MAPK) were performed to detect their activities in hPSCs (Figure 5A). We found that ILG inhibited the phosphorylated activation of both ERK1/2 and JNK1/2, but not p38 MAPK, in a dose‐dependent manner (Figure 5A, left panel). Meanwhile, the phosphorylation of c‐Jun, the downstream co‐transcriptional factor, was also restrained by ILG. Next, we used TGF‐β1 to induce high level activation of hPSCs and found that the phosphorylation of above three MAPKs and c‐Jun were obviously increased (Figure 5A, right panel, line 2), which is consistent with previous studies. In this context, ILG administration could still significantly restrain the phosphorylation of ERK1/2, JNK1/2 and c‐Jun, but not p38 MAPK. IF assays further confirmed the regulation of c‐Jun activity by ILG and TGF‐β1, respectively, or together (Figure S2). All these data suggested that ILG may inhibit the activation of hPSCs via suppressing the activities of ERK1/2 and JNK1/2, blocking downstream signal transduction.

The activity of MAPKs is modulated by upstream signals (kinases) and/or negative regulators (phosphatases). From previous RNA‐seq data, the negative regulation of MAPK activity and the PDGFR signalling pathway were obviously regulated by ILG (Figure 4C‐E), which were later validated in our in vitro assays in hPSCs (Figure 5A). We next evaluated the effects of ILG on the expression of DUSPs by qRT‐PCR and WB. As shown in Figure 5B, ILG treatment significantly increased the mRNA level of DUSP5 and DUSP10 dose‐dependently and rescued the inhibitory effect of TGF‐β1, while DUSP1/MKP1 (interact and dephosphorylate all three MAPKs) and DUSP16/MKP7 (binds and inactivates JNK1/2 and p38 MAPK) were hardly affected. WB and IF assays further confirmed that ILG up‐regulated the expression of DUSP5 and DUSP10 at protein level (Figure 5D and E, Figure S2).

Collectively, as depicted in Figure 5F, it may be both the inhibition of upstream PDGFR activity and elevated expression of inhibitory DUSP5 and DUSP10 combined contribute to the negative regulation of ERK1/2 and JNK1/2 activities by ILG, which finally lead to its inhibitory effect on the activation of PSCs and pancreatic fibrosis.

### 3.7 ILG restrains the pancreatic infiltration and M1 polarization of macrophages

We next sought to investigate the effect of ILG on the immune responses in CP. As reported in previous studies, the tissue infiltration of macrophage occurs in two distinct ways: recruitment of monocyte precursors and proliferation of resident cells.^15^, ^16^ In our study, chemokines and cytokines produced by activated hPSCs were all suppressed by ILG (Figure 2G), indicating that ILG might restrain the pancreatic infiltration of macrophages by down‐regulation of local production of pro‐inflammatory cytokines and chemokines.

**FIGURE 6:**
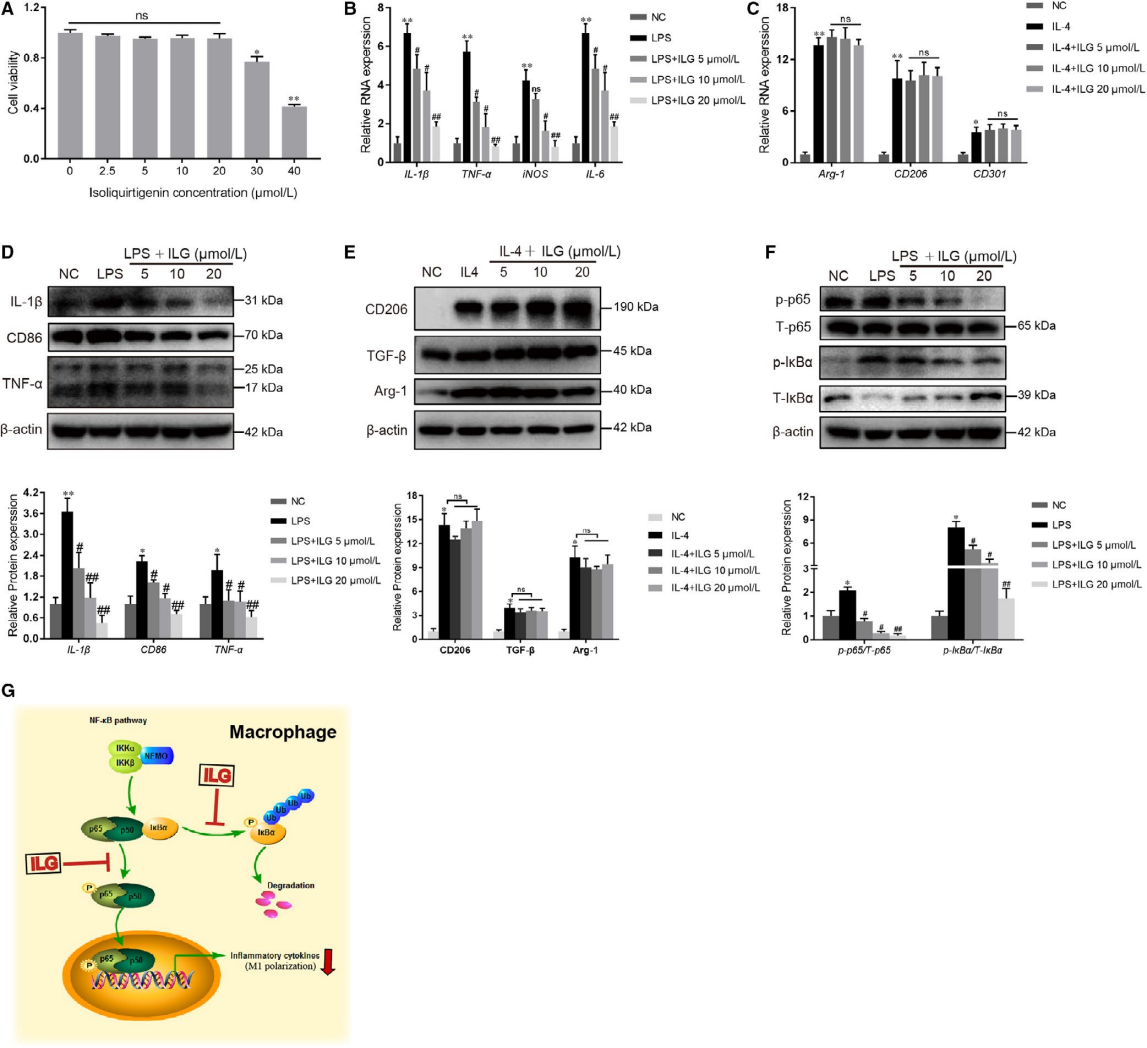
ILG restrains the M1 polarization of macrophages through NF‐κB pathway. (A) RAW 264.7 cells were treated with different doses of ILG for 24 h, and then, the cell viability was measured by CCK‐8. (b and e) The effects of ILG on macrophages polarization were evaluated. The mRNA and protein expression level of M1‐type markers (B and D, 500 ng/mL LPS pre‐induction for 1 h) and M2‐type markers (C and E, 20 ng/ml IL‐4 pre‐induction for 24 h) were determined by qRT‐PCR and WB, respectively. (F) RAW 264.7 cells were pre‐treated with ILG for 1 h and subsequently stimulated with LPS for 6 h. The effects of ILG on the NF‐κB signalling pathway (p‐p65 and p65, p‐IκBα and IκBα) were detected by WB assays. The band signals were assessed using GeneTools software and statistically presented in the histogram below. All data were presented as mean ± SD of at least three independent experiments. *P < .05, **P < .01 compared with the NC group; #*P* < .05, ##*P* < .01 compared with the LPS group; ns: *P* ＞ .05. (G) A schematic diagram illustrating the underlying mechanism by which ILG inhibits the NF‐κB signalling and the M1 polarization of macrophages

Besides, the M1/M2 polarization of macrophages also plays important roles in the progression of CP. Thus, we examined the effect of ILG on cell viability and polarization of macrophages in RAW 264.7 cells (Figure 6). We used LPS (500 ng/ml, Figure 6B and D) or IL‐4 (20 ng/ml, Figure 6C and E) to induce the M1 or M2 polarization, respectively. Upon induction, the mRNA and protein levels of the M1 or M2 polarization associated genes were all significantly increased. The mRNA transcription and protein level of those M1 associated genes, including *IL‐1β*, *TNF‐α*, *iNOS*, *IL‐6* and *CD86*, were all inhibited by ILG dose‐dependently, while the M2 associated genes like *Arg‐1*, *CD206*, *CD301* and *TGF‐β1* were hardly affected (Figure 6B‐E).

Further mechanism research suggested that ILG inhibited the M1 polarization of RAW 264.7 cells via attenuation of the NF‐κB signalling pathway, and this was associated with a decrease in the phosphorylation of IκBα and p65 subunit, and blocking of the subsequent nuclear translocation of p65 (Figure 6F and G). Since the M1 polarization is usually thought to be related to pro‐inflammatory responses in physiological conditions, our results suggesting another possible mechanism that ILG might restrain the pancreatic infiltration of macrophages by inhibiting its M1 polarization.

## 4 DISCUSSION

Due to the limited availability of tissues obtained from surgery, studies on pathogenic mechanism of pancreatic fibrosis in human CP are restricted to a large extent. Thus, animal models have been useful to investigate the initiation and progression of CP and to test new treatments, despite their limitation in recapitulating all aspects of human disease. The Cae‐induced murine pancreatitis has been widely accepted as a clinically relevant model for recurrent acute necrotizing pancreatitis. Based on this model, we found that ILG exerts a significant inhibitory effect on the pancreatic fibrosis and inflammation by reducing both the activation of PSCs and pancreatic infiltration of macrophages. Further transcriptomic analysis and validation experiments in hPSCs indicated that ILG inhibited the activation of PSCs via inhibiting the PDGFR signalling and negative regulation of the MAPKs activities.

The DUSPs, in particular those DUSP‐MKPs, have been identified as immediate early genes whose protein expression is rapidly induced by oxidative stress, growth factors and heat shock.^32^, ^33^, ^34^ It has been suggested that these inducible expressed DUSPs might be key players in feedback loop mechanism, since they can terminate the signal transduction mediated by MAPKs.^35^, ^36^ In addition, the expression of DUSP5 is also reported to be regulated through epigenetic events involving promoter hypermethylation ^37^ and LncRNA H19.^35^ Besides, the protein expression and phosphatase activity of these DUSPs are also regulated by post‐transcriptional modifications and protein stability.^31^, ^38^, ^39^ It is still not clear how the expression of DUSP5 and DUSP10 is regulated by ILG, which might involve both transcriptional and post‐transcriptional mechanisms.

Previous studies have proposed that macrophages are the predominant inflammatory cells infiltrated in CP and have a close proximity to PSCs. In our murine CP model, the infiltration of macrophages was confirmed and was suppressed by ILG treatment. Further in vitro studies in RAW 264.7 cells suggested that ILG inhibited the M1 polarization of macrophage. To our surprise, ILG hardly affected the M2 polarization of macrophage, which is reported to be pro‐fibrogenic and is the main kind macrophage in CP. Considering the CP animal model is established by induction of repeated and continuous acute injury to the pancreas (AP) to drive the occurrence of chronic pancreatic inflammation and fibrosis, ILG may exert its inhibitory effect through suppressing each acute inflammatory response, including the inhibition of PSCs activation, the expression and secretion of pro‐inflammatory cytokines that contribute to proliferation or recruitment of local macrophage or monocyte from blood, and the M1 polarization of macrophage.

ILG is an abundant dietary flavonoid, which is an important constituent in licorice. Licorice has been used for more than 4 millennia as a flavouring agent in foods, tobacco, beverages, and to treat individuals with gastric or duodenal ulcers, coughs, sore throats and allergies.^40^, ^41^ Since natural extracts are usually low in toxicity and are well tolerated in humans, increasing attention has been given to seeking anti‐fibrotic and anti‐inflammatory agents from natural sources. Thus, ILG might be a promising therapeutic agent for CP in clinical practice. Of course, follow‐up studies are needed to investigate the effect of ILG on other cell types in pancreas, such as acinar cells, islet cells and other immune cells (like CD4^+^ T cells), in order to further evaluate its clinical value for CP.

## 5 CONFLICT OF INTEREST

The authors confirm that there are no conflicts of interest.

## AUTHOR CONTRIBUTION

Li‐juan Wang: Conceptualization (lead); Data curation (equal); Formal analysis (equal); Funding acquisition (supporting); Investigation (equal); Methodology (lead); Project administration (equal); Supervision (equal); Writing‐original draft (lead); Writing‐review & editing (equal). Lin He: Data curation (equal); Formal analysis (equal); Investigation (equal); Methodology (equal); Software (equal); Validation (equal); Writing‐original draft (equal). Lu Hao: Data curation (equal); Formal analysis (equal); Investigation (equal); Software (equal). Hong‐lei Guo: Formal analysis (supporting); Methodology (supporting); Software (supporting); Validation (supporting). Xiang‐peng Zeng: Formal analysis (supporting); Investigation (supporting); Methodology (supporting); Software (supporting). Ya‐wei Bi: Investigation (supporting); Validation (supporting). Guo‐tao Lu: Conceptualization (supporting); Methodology (supporting); Resources (supporting); Supervision (equal); Validation (supporting). Zhao‐shen Li: Conceptualization (supporting); Funding acquisition (equal); Project administration (equal); Resources (equal); Supervision (equal); Writing‐review & editing (equal). Liang‐hao Hu: Conceptualization (supporting); Funding acquisition (lead); Project administration (equal); Resources (equal); Supervision (lead); Writing‐review & editing (equal). All authors revised the manuscript critically and approved the final version.

## Supporting information

Fig S1

Fig S2

Table S1

Table S2

## Data Availability

The datasets used and/or analysed during the current study are available from the corresponding author on reasonable request.

## References

[1] Kleeff J , Whitcomb DC , Shimosegawa T , et al. Chronic pancreatitis. Nature Reviews Disease Primers. 2017;3:17060. 10.1038/nrdp.2017.60 28880010

[2] Li B‐R , Liao Z , Du T‐T , et al. Risk factors for complications of pancreatic extracorporeal shock wave lithotripsy. Endoscopy. 2014;46:1092‐1100. 25251205 10.1055/s-0034-1377753

[3] Wang D , Bi Y‐W , Ji J‐T , et al. Extracorporeal shock wave lithotripsy is safe and effective for pediatric patients with chronic pancreatitis. Endoscopy. 2017;49:447‐455. 28403504 10.1055/s-0043-104527 PMC6298383

[4] Xue R , Jia K , Wang J , et al. A rising star in pancreatic diseases: pancreatic stellate cells. Front Physiol. 2018;9:754. 29967585 10.3389/fphys.2018.00754 PMC6015921

[5] Plotnikov A , Zehorai E , Procaccia S , et al. The MAPK cascades: signaling components, nuclear roles and mechanisms of nuclear translocation. Biochem Biophys Acta. 2011;1813:1619‐1633. 21167873 10.1016/j.bbamcr.2010.12.012

[6] Dhanasekaran DN , Reddy EP . JNK signaling in apoptosis. Oncogene. 2008;27:6245‐6251. 18931691 10.1038/onc.2008.301 PMC3063296

[7] Masamune A , Kikuta K , Satoh M , et al. Differential roles of signaling pathways for proliferation and migration of rat pancreatic stellate cells. The Tohoku journal of experimental medicine. 2003;199:69‐84. 12705352 10.1620/tjem.199.69

[8] Xu XF , Liu F , Xin JQ , et al. Respective roles of the mitogen‐activated protein kinase (MAPK) family members in pancreatic stellate cell activation induced by transforming growth factor‐beta1 (TGF‐beta1). Biochem Biophys Res Comm. 2018;501:365‐373. 29705706 10.1016/j.bbrc.2018.04.176

[9] Jaster R , Sparmann G , Emmrich J , et al. Extracellular signal regulated kinases are key mediators of mitogenic signals in rat pancreatic stellate cells. Gut. 2002;51:579‐584. 12235084 10.1136/gut.51.4.579 PMC1773393

[10] Masamune A , Kikuta K , Suzuki N , et al. A c‐Jun NH2‐terminal kinase inhibitor SP600125 (anthra[1,9‐cd]pyrazole‐6 (2H)‐one) blocks activation of pancreatic stellate cells. The Journal of pharmacology and experimental therapeutics. 2004;310:520‐527. 15056726 10.1124/jpet.104.067280

[11] Masamune A , Satoh M , Kikuta K , et al. Inhibition of p38 mitogen‐activated protein kinase blocks activation of rat pancreatic stellate cells. The Journal of pharmacology and experimental therapeutics. 2003;304:8‐14. 12490569 10.1124/jpet.102.040287

[12] Wynn TA , Barron L . Macrophages: master regulators of inflammation and fibrosis. Semin Liver Dis. 2010;30:245‐257. 20665377 10.1055/s-0030-1255354 PMC2924662

[13] Van Dyken SJ , Locksley RM . Interleukin‐4‐ and interleukin‐13‐mediated alternatively activated macrophages: roles in homeostasis and disease. Annu Rev Immunol. 2013;31:317‐343. 23298208 10.1146/annurev-immunol-032712-095906 PMC3606684

[14] Xue J , Sharma V , Hsieh MH , et al. Alternatively activated macrophages promote pancreatic fibrosis in chronic pancreatitis. Nat Commun. 2015;6:7158. 25981357 10.1038/ncomms8158 PMC4632846

[15] Saeki K , Kanai T , Nakano M , et al. CCL2‐induced migration and SOCS3‐mediated activation of macrophages are involved in cerulein‐induced pancreatitis in mice. Gastroenterology. 2012;142(1010–1020):e1019. 10.1053/j.gastro.2011.12.054 22248664

[16] Jenkins SJ , Ruckerl D , Thomas GD , et al. IL‐4 directly signals tissue‐resident macrophages to proliferate beyond homeostatic levels controlled by CSF‐1. J Exp Med. 2013;210:2477‐2491. 24101381 10.1084/jem.20121999 PMC3804948

[17] Wynn TA , Ramalingam TR . Mechanisms of fibrosis: therapeutic translation for fibrotic disease. Nat Med. 2012;18:1028‐1040. 22772564 10.1038/nm.2807 PMC3405917

[18] Jin XY , Sohn DH , Lee SH . Isoliquiritigenin suppresses tumor necrosis factor‐alpha‐induced inflammation via peroxisome proliferator‐activated receptor‐gamma in intestinal epithelial cells. Arch Pharm Res. 2016;39:1465‐1471. 27539609 10.1007/s12272-016-0805-x

[19] Chi JH , Seo GS , Cheon JH , et al. Isoliquiritigenin inhibits TNF‐alpha‐induced release of high‐mobility group box 1 through activation of HDAC in human intestinal epithelial HT‐29 cells. Eur J Pharmacol. 2017;796:101‐109. 28012970 10.1016/j.ejphar.2016.12.026

[20] Na A‐Y , Yang E‐J , Jeon JM , et al. Protective effect of isoliquiritigenin against ethanol‐induced hepatic steatosis by regulating the SIRT1‐AMPK pathway. Toxicological Research. 2018;34:23‐29. 29371998 10.5487/TR.2018.34.1.023 PMC5776912

[21] Watanabe Y , Nagai Y , Honda H , et al. Isoliquiritigenin attenuates adipose tissue inflammation in vitro and adipose tissue fibrosis through inhibition of innate immune responses in mice. Sci Rep. 2016;6:23097. 26975571 10.1038/srep23097 PMC4791553

[22] Wu S , Xue J , Yang Y , et al. Isoliquiritigenin inhibits interferon‐gamma‐inducible genes expression in hepatocytes through down‐regulating activation of JAK1/STAT1, IRF3/MyD88, ERK/MAPK, JNK/MAPK and PI3K/Akt signaling pathways. Cell Physiol Biochem. 2015;37:501‐514. 26315837 10.1159/000430372

[23] Liu X , Zhu Q , Zhang M , et al. Isoliquiritigenin ameliorates acute pancreatitis in mice via inhibition of oxidative stress and modulation of the Nrf2/HO‐1 pathway. Oxidative Med Cell Long. 2018;2018:7161592. 10.1155/2018/7161592 PMC5944199 29854090

[24] Zeng X‐P , Wang L‐J , Guo H‐L , et al. Dasatinib ameliorates chronic pancreatitis induced by caerulein via anti‐fibrotic and anti‐inflammatory mechanism. Pharmacol Res. 2019;147:104357. 31356863 10.1016/j.phrs.2019.104357

[25] Zhang G‐X , Wang M‐X , Nie W , et al. P2X7R blockade prevents NLRP3 inflammasome activation and pancreatic fibrosis in a mouse model of chronic pancreatitis. Pancreas. 2017;46:1327‐1335. 28930866 10.1097/MPA.0000000000000928

[26] Hu Y , Wan R , Yu GE , et al. Imbalance of Wnt/Dkk negative feedback promotes persistent activation of pancreatic stellate cells in chronic pancreatitis. PLoS One. 2014;9:e95145. 24747916 10.1371/journal.pone.0095145 PMC3991593

[27] Phillips PA , Wu MJ , Kumar RK , et al. Cell migration: a novel aspect of pancreatic stellate cell biology. Gut. 2003;52:677‐682. 12692052 10.1136/gut.52.5.677 PMC1773645

[28] Woo S , Lee S , Ko G , et al. Isoliquiritigenin inhibits cell proliferation by a heme oxygenase‐dependent pathway in rat hepatic stellate cells. Planta Med. 2008;74:834‐839. 18563666 10.1055/s-2008-1074555

[29] Huang CY , Tan TH . DUSPs, to MAP kinases and beyond. Cell Biosci. 2012;2:24. 22769588 10.1186/2045-3701-2-24 PMC3406950

[30] Caunt CJ , Keyse SM . Dual‐specificity MAP kinase phosphatases (MKPs): shaping the outcome of MAP kinase signalling. FEBS J. 2013;280:489‐504. 22812510 10.1111/j.1742-4658.2012.08716.x PMC3594966

[31] Chen HF , Chuang HC , Tan TH . Regulation of dual‐specificity phosphatase (dusp) ubiquitination and protein Stability. Intern J Mol Sci. 2019;20(11):2668. 10.3390/ijms20112668 PMC6600639 31151270

[32] Keyse SM , Emslie EA . Oxidative stress and heat shock induce a human gene encoding a protein‐tyrosine phosphatase. Nature. 1992;359:644‐647. 1406996 10.1038/359644a0

[33] Charles CH , Sun H , Lau LF , et al. The growth factor‐inducible immediate‐early gene 3CH134 encodes a protein‐tyrosine‐phosphatase. Proc Natl Acad Sci. 1993;90(11):5292‐5296. 8389479 10.1073/pnas.90.11.5292 PMC46702

[34] Zhang T , Roberson MS . Role of MAP kinase phosphatases in GnRH‐dependent activation of MAP kinases. J Mol Endocrinol. 2006;36:41‐50. 16461925 10.1677/jme.1.01881

[35] Tao H , Cao W , Yang J‐J , et al. Long noncoding RNA H19 controls DUSP5/ERK1/2 axis in cardiac fibroblast proliferation and fibrosis. Cardiovas Pathol.. 2016;25:381‐389. 10.1016/j.carpath.2016.05.005 27318893

[36] Habibian JS , Jefic M , Bagchi RA , et al. DUSP5 functions as a feedback regulator of TNFalpha‐induced ERK1/2 dephosphorylation and inflammatory gene expression in adipocytes. Sci Rep. 2017;7:12879. 29018280 10.1038/s41598-017-12861-y PMC5635013

[37] Shin SH , Park SY , Kang GH . Down‐regulation of dual‐specificity phosphatase 5 in gastric cancer by promoter CpG island hypermethylation and its potential role in carcinogenesis. Am J Pathol. 2013;182:1275‐1285. 23402999 10.1016/j.ajpath.2013.01.004

[38] Kucharska A , Rushworth LK , Staples C , et al. Regulation of the inducible nuclear dual‐specificity phosphatase DUSP5 by ERK MAPK. Cell Signal. 2009;21:1794‐1805. 19666109 10.1016/j.cellsig.2009.07.015

[39] Benavides‐Serrato A , Anderson L , Holmes B , et al. mTORC2 modulates feedback regulation of p38 MAPK activity via DUSP10/MKP5 to confer differential responses to PP242 in glioblastoma. Genes & Cancer. 2014;5:393‐406. 25568665 10.18632/genesandcancer.41 PMC4279437

[40] Fintelmann V . Modern phytotherapy and its uses in gastrointestinal conditions. Planta Med. 1991;57:S48‐52. 10.1055/s-2006-960229 17226223

[41] Haggag EG , Abou‐Moustafa MA , Boucher W , et al. The effect of a herbal water‐extract on histamine release from mast cells and on allergic asthma. Journal of herbal pharmacotherapy. 2003;3:41‐54. 15277119

