# Supplementary figures and images for: Isoliquiritigenin ameliorates caerulein‐induced chronic pancreatitis by inhibiting the activation of PSCs and pancreatic infiltration of macrophages
Source: J Cell Mol Med. 2020 Jul 17;24(17):9667–81. doi: 10.1111/jcmm.15498 (PMC7520303; doi:10.1111/jcmm.15498)

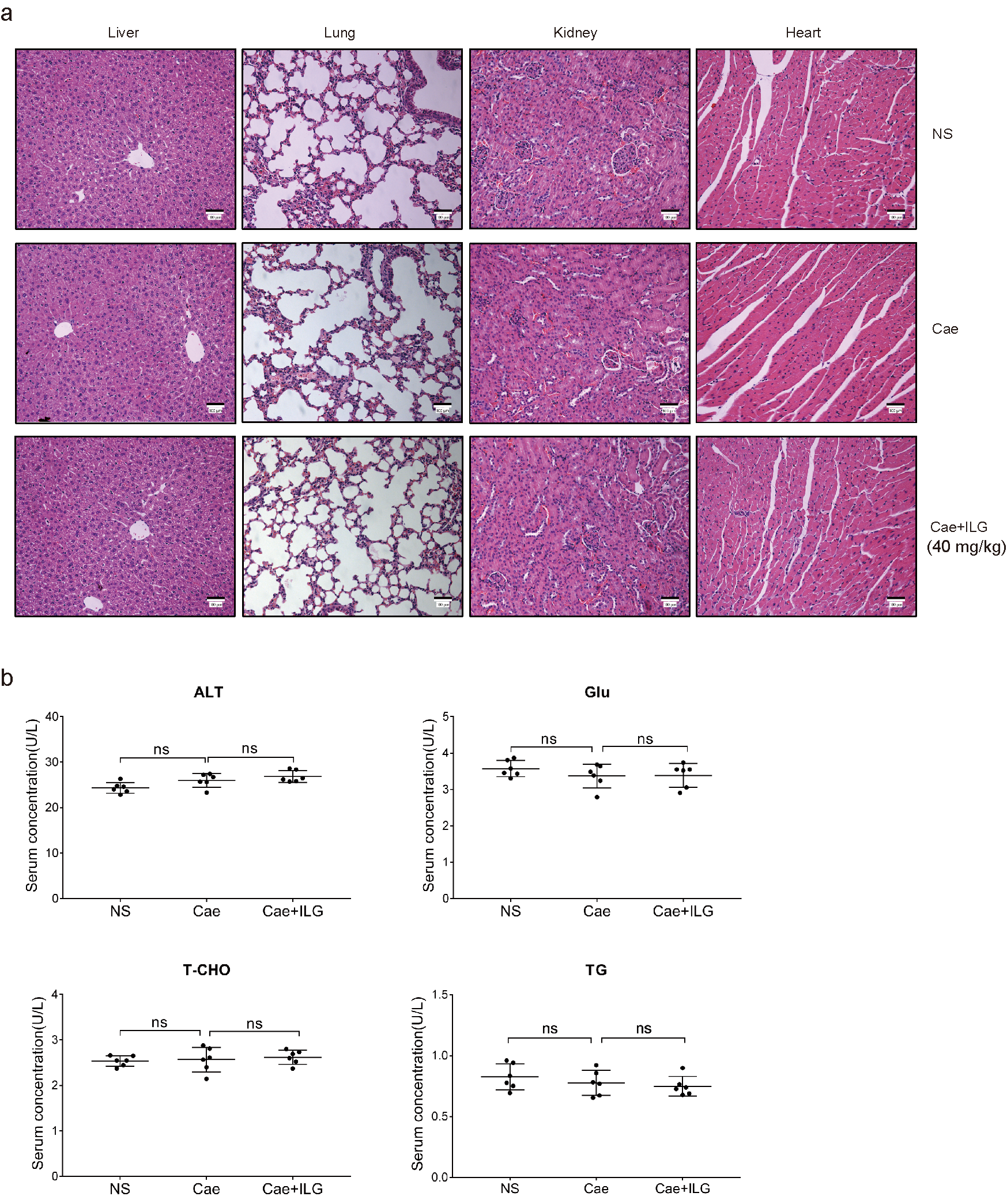

Supplement: Supplementary file 1 — Fig S1 [file JCMM-24-9667-s001.tif]

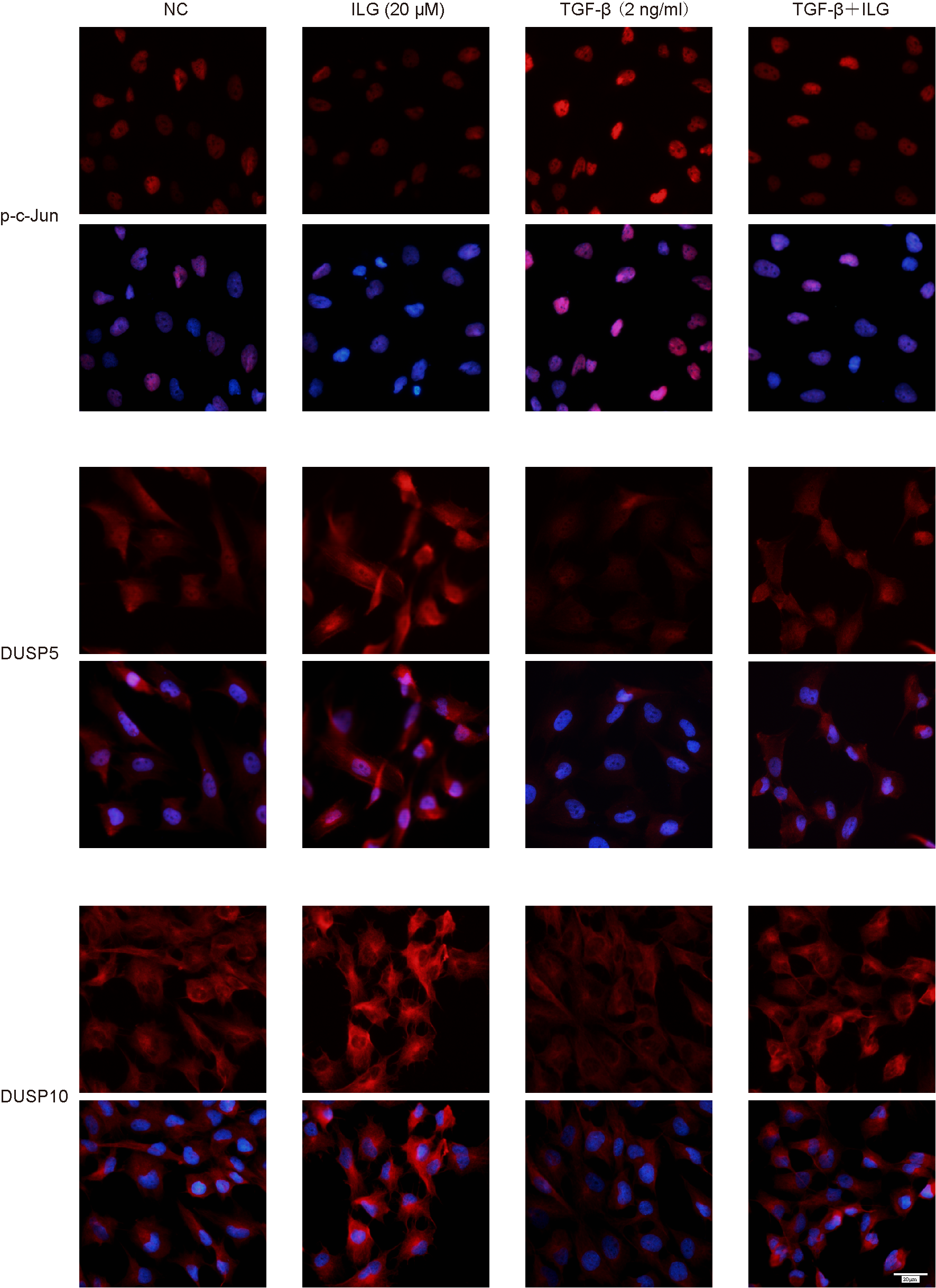

Supplement: Supplementary file 2 — Fig S2 [file JCMM-24-9667-s002.tif]
