# Supplementary material for: Isoliquiritigenin ameliorates caerulein‐induced chronic pancreatitis by inhibiting the activation of PSCs and pancreatic infiltration of macrophages
Source: J Cell Mol Med. 2020 Jul 17;24(17):9667–81. doi: 10.1111/jcmm.15498 (PMC7520303; doi:10.1111/jcmm.15498)
Supplement: Supplementary file 3 — Table S1 [file JCMM-24-9667-s003.doc]

**Table 1**. Primer Sequences Used for qRT-PCR. (h: human; m: mouse)

| Gene | Forward sequence (5’-3’) | Reverse sequence (5’-3’) |
| --- | --- | --- |
| hα-SMA  hFN  hCOL-1  hDUSP1  hDUSP5  hDUSP10  hDUSP16  hIL1-β  hTNF-α  hIL-6  hCCL2  hCXCL1  hCCL5  hGAPDH | ACGAAGCGGCTGGCAACCTCAT  CCCAATTGAGTGCTTCATGCC  CAGCCGCTTCACCTACAGC  TTTGAGGGTCACTACCAG  CTGAGTGTTGCGTGGA  AGGTCATAGGCATCGTT  AGAATGGGATTGGTTATGTG  AGCTACGAATCTCCGACCAC  CCCATGTTGTAGCAAACCCTC  CCAGAGCTGTGCAGATGAGT  GATCTCAGTGCAGAGGCTCG  ATTCACCCCAAGAACATCCA  CTGCTGCTTTGCCTACATTG  GACAGTCAGCCGCATCTTCT | CCCTCGGCCCTCATCTCTACATCA  AACTCCCAGGGTGATGCTTG  TTTTGTATTCAATCACTGTCTTGCC  GAGATGATGCTTCGCC  AGTCTATTGCTTCTTGAAAGT  CTGAACATCGGCTACG  TGTAGGCGATAGCGATG  CGTTATCCCATGTGTCGAAGAA  TGAGGTACAGGCCCTCTGAT  CTGCAGCCACTGGTTCTGT  TTTGCTTGTCCAGGTGGTCC  CACCAGTGAGCTTCCTCCTC  TTTCTTCTCTGGGTTGGCAC  GCGCCCAATACGACCAAATC |
| mα-SMA  mFN  mCOL-1  mCCL2  mCD68  mF4/80  mIL1-β  mTNF-α  miNOS  mIL-6  mArg-1  mCD206  mCD301  mGapdh | GCCAGTCGCTGTCAGGAACCC  GCCTGAGGTGGACCCCGCTA  CGCCATCAAGGTCTACTG  CAAGAGGATCACCAGCAGCA  GGTCCCTATGTCTCCAACTGC  GTGAAGATCCGCGACTGGTA  ACGAAGCGGCTGGCAACCTC  CCAAAGGGATGAGAAGTTCC  GAGGCCCAGGAGGAGAGAGTCC AGTTGCCTTCTTGGGACTGA  AGACCACAGTCTGGCAGTTG  TGATTACGAGCAGTGGAAGC  ACTGAGTTCCTGCCTCTGGT  GGTCGGTGTGAACGGATTTG | CCAGCGAAGCCGGCCTTACA  GGGCCCAAGTGACCCGCATC  ACGGGAATCCATCGGTC  TGCTTGAGGTGGTTGTGGAA  CTTGATGGCAATCGTCTTCAGA  TGTTCTGTCTCAAACTTGGTTCT  CCCTCGGCCCTCATCTCTACATCA  CTCCACTTGGTGGTTTGCTA  TCCATGCAGACAACCTTGGTGTTG  TCCACGATTTCCCAGAGAAC  CCACCCAAATGACACATAGG  GTTCACCGTAAGCCCAATTT  ATCTGGGACCAAGGAGAGTG  TGTAGACCATGTAGTTGAGGTCA |
