# Supplementary material for: Isoliquiritigenin ameliorates caerulein‐induced chronic pancreatitis by inhibiting the activation of PSCs and pancreatic infiltration of macrophages
Source: J Cell Mol Med. 2020 Jul 17;24(17):9667–81. doi: 10.1111/jcmm.15498 (PMC7520303; doi:10.1111/jcmm.15498)
Supplement: Supplementary file 4 — Table S2 [file JCMM-24-9667-s004.doc]

**Table 2. Primary Antibody Used in This Study.**

| Antibody | Company | Dilution |
| --- | --- | --- |
| Collagen I | Abcam (ab34710) | 1:200 |
| CD68 | Abcam (ab955) | 1:200 |
| CD86 | Abcam (ab53004) | 1:500 |
| CD206 | Abcam (ab64693) | 1:500 |
| α-SMA | Cell Signaling Technology (#19245) | 1:1000 |
| Fibronectin | Santa Cruz Biotechnology (sc-8422) | 1:500 |
| CTGF | Cell Signaling Technology (#86641) | 1:1000 |
| Cyclin D1 (92G2) | Cell Signaling Technology (#2978) | 1:1000 |
| c-Myc (D84C12) | Cell Signaling Technology (#5605) | 1:1000 |
| CleavedCaspase-3 (Asp175) | Cell Signaling Technology (#9664) | 1:1000 |
| β-Catenin | Abcam (ab32572) | 1:2000 |
| active β-Catenin | Cell Signaling Technology (#8814) | 1:1000 |
| JNK1/2 | Cell Signaling Technology (#9252) | 1:1000 |
| p-JNK1/2 | Cell Signaling Technology (#4668) | 1:500 |
| PDGFR-β | Cell Signaling Technology (#3169) | 1:1000 |
| p-PDGFR-β | Cell Signaling Technology (#4549) | 1:1000 |
| p38 MAPK | Cell Signaling Technology (#8690) | 1:1000 |
| p-p38 MAPK | Cell Signaling Technology (#4511) | 1:1000 |
| Erk1/2 | Cell Signaling Technology (#4695) | 1:1000 |
| p-Erk1/2 | Cell Signaling Technology (#4370) | 1:2000 |
| c-Jun | Cell Signaling Technology (#9165) | 1:1000 |
| p-c-Jun | Cell Signaling Technology (#3270) | 1:500 |
| IL-1β | Cell Signaling Technology (#12242) | 1:500 |
| TNF-α | Cell Signaling Technology (#3707) | 1:500 |
| Arg-1 | Cell Signaling Technology (#93668) | 1:1000 |
| NF-κB p65 | Cell Signaling Technology (#8242) | 1:1000 |
| p-NF-κB p65 | Cell Signaling Technology (#3033) | 1:1000 |
| TGF-β | Cell Signaling Technology (#3711) | 1:1000 |
| IκBα | Cell Signaling Technology (#9246) | 1:1000 |
| p-IκBα | Cell Signaling Technology (#4814) | 1:1000 |
| DUSP5 | Santa Cruz Biotechnology (sc-515667) | 1:500 |
| DUSP10 | Santa Cruz Biotechnology (sc-374276) | 1:500 |
| β-actin | Cell Signaling Technology (#4970) | 1:5000 |
